# Thank You for Being Here: Insights From Rural Oncology Practice

**DOI:** 10.1200/OP.23.00760

**Published:** 2024-02-14

**Authors:** Wade T. Swenson

**Affiliations:** ^1^ Lakewood Health System, Staples, MN; ^2^ Department of Internal Medicine, University of North Dakota School of Medicine, Fargo, ND

## Abstract

Rural oncologist reflects on 20 years of providing vital cancer care in underserved areas.

Thank you for being here. A simple phrase, often spoken in passing, but in the context of a rural oncology clinic, it takes on a profound meaning. It is an acknowledgment from patients who understand the scarcity of specialized care in their rural communities. This simple phrase encapsulates the appreciation for accessible, compassionate care in areas where it is most needed. I have encountered unique challenges and rewards as a medical oncologist practicing in a rural setting for nearly 2 decades. The disparity in health care access is particularly stark in oncology. Interventions such as advanced diagnostics, specialized surgeries, and radiation therapy require resources often unavailable in smaller, rural hospitals. In a town of just 2,900 people, every patient visit becomes more than a medical encounter; it is a significant event for the community, laden with stories and shared experiences.

My journey into rural oncology was not planned. During fellowship, I had envisioned a career in a suburban midwest city, but an opportunity in a rural community close to my family changed my trajectory. A chance to work alongside a semi-retired oncologist I admired greatly influenced my decision. Two decades later, I find myself deeply committed to rural cancer care. Patients in rural Minnesota often travel long distances for care, adding stress to their already challenging situations. Our local hospitals and clinics strive to provide essential services despite financial constraints. When patients say thank you for being here, they are acknowledging the care they receive and our commitment to the community.

Our clinic's unique assets include a board-certified palliative care physician, a rarity in a rural setting, and ample access to mental health care services within our system. We also employ oncology case managers, financial navigators, and patient navigators who provide a rural oncology home for patients and a point of contact for patients throughout their cancer journey, whether in our health system or receiving care at a regional tertiary system. We work with oral oncolytic navigators at a specialty pharmacy. These team members are crucial in providing compassionate, personalized community-based care. We refer patients for radiation oncology, and many patients travel 2 hours round-trip for daily radiotherapy. Our journey with clinical trials has been fraught with challenges. Initially, collaborations with regional partners were our primary method, but the extensive clinical trial infrastructure required forced a transition to digital platforms for matching genomic tumor profiles with clinical trials. We have a locally based tumor board that involves specialists from various regional systems. This collaborative approach is vital in managing complex cases and making informed decisions.

In a rural oncology clinic, a common scenario involves older patients being accompanied by their adult children, who often live in distant cities. The children typically arrive having researched our oncology team online, seeking the best possible care for their parents. They journey to our clinic for the initial oncology visit, filled with apprehension about the quality of care their parents will receive in a rural setting. This dynamic reflects a broader trend in health care, where patients and their families vet providers and treatment options online. In rural areas, where specialized medical services are perceived to be limited, this scrutiny can be even more intense. Adult children, who often have access to more health care options in urban areas, are naturally concerned about the standards of care available to their parents in a rural clinic.

On arriving at our clinic, these families are often pleasantly surprised. They find a team of dedicated and skilled professionals who are not only knowledgeable but also deeply compassionate. Our team, aware of the anxieties and expectations of both patients and their families, makes it a priority to build trust and demonstrate our commitment to providing high-quality care. One of the most rewarding aspects of my work in rural oncology is seeing the relief and reassurance on the faces of these family members. After consultations, where we discuss treatment plans and address any concerns, they often express their gratitude. They realize that their parents are indeed in good hands and that the necessity for frequent trips to regional centers for the majority of their care is not as critical as they had anticipated. This reassurance is crucial. It not only eases the burden on the patients and their families but also reinforces the importance of quality rural health care. It highlights that despite geographical and resource challenges, rural health care providers are capable of delivering care that meets urban standards.

Overall, these encounters underscore a vital aspect of rural oncology: the ability to provide high-quality cancer care close to home, reducing the need for patients to travel long distances for treatment. It is about building relationships, understanding each patient's story, and providing care that encompasses not just their physical health but their emotional and social well-being. It is about being a consistent presence in a journey that can be incredibly daunting. Every thank you for being here either from a patient or family member is a testament to the importance of rural health care. It underscores the value of our presence in these rural communities. These expressions of gratitude are not just courtesies, they are acknowledgments of the partnership in the journey toward healing. They remind us that our role is not only to provide medical care but to offer hope, support, and understanding.

